# PET measurement of cyclooxygenase-2 using a novel radioligand: upregulation in primate neuroinflammation and first-in-human study

**DOI:** 10.1186/s12974-020-01804-6

**Published:** 2020-05-02

**Authors:** Stal Shrestha, Min-Jeong Kim, Mark Eldridge, Michael L. Lehmann, Michael Frankland, Jeih-San Liow, Zu-Xi Yu, Michelle Cortes-Salva, Sanjay Telu, Ioline D. Henter, Evan Gallagher, Jae-Hoon Lee, J. Megan Fredericks, Chelsie Poffenberger, George Tye, Yanira Ruiz-Perdomo, Fernanda Juarez Anaya, Jose A. Montero Santamaria, Robert L. Gladding, Sami S. Zoghbi, Masahiro Fujita, James D. Katz, Victor W. Pike, Robert B. Innis

**Affiliations:** 1 National Institute of Mental Health Intramural Research Program, Bethesda, MD USA; 2 Medical Scientist Training Program, University of Miami Miller School of Medicine, Miami, FL USA; 3 Molecular Imaging Branch, National Institute of Mental Health, 10 Center Drive, Bldg. 10, Rm B1D43, Bethesda, MD 20892-1026 USA; 4 National Heart, Lung, and Blood Institute Intramural Research Program, Bethesda, MD USA; 5 National Institute of Arthritis and Musculoskeletal and Skin Diseases, Bethesda, MD USA

**Keywords:** Cyclooxygenase 2, Cyclooxygenase 1, Positron emission tomography, Inflammation, Lipopolysaccharide, Rheumatoid arthritis

## Abstract

**Background:**

Cyclooxygenase-2 (COX-2), which is rapidly upregulated by inflammation, is a key enzyme catalyzing the rate-limiting step in the synthesis of several inflammatory prostanoids. Successful positron emission tomography (PET) radioligand imaging of COX-2 in vivo could be a potentially powerful tool for assessing inflammatory response in the brain and periphery. To date, however, the development of PET radioligands for COX-2 has had limited success.

**Methods:**

The novel PET tracer [^11^C]MC1 was used to examine COX-2 expression [1] in the brains of four rhesus macaques at baseline and after injection of the inflammogen lipopolysaccharide (LPS) into the right putamen, and [2] in the joints of two human participants with rheumatoid arthritis and two healthy individuals. In the primate study, two monkeys had one LPS injection, and two monkeys had a second injection 33 and 44 days, respectively, after the first LPS injection. As a comparator, COX-1 expression was measured using [^11^C]PS13.

**Results:**

COX-2 binding, expressed as the ratio of specific to nondisplaceable uptake (*BP*_ND_) of [^11^C]MC1, increased on day 1 post-LPS injection; no such increase in COX-1 expression, measured using [^11^C]PS13, was observed. The day after the second LPS injection, a brain lesion (~ 0.5 cm in diameter) with high COX-2 density and high *BP*_ND_ (1.8) was observed. Postmortem brain analysis at the gene transcript or protein level confirmed in vivo PET results. An incidental finding in an unrelated monkey found a line of COX-2 positivity along an incision in skull muscle, demonstrating that [^11^C]MC1 can localize inflammation peripheral to the brain. In patients with rheumatoid arthritis, [^11^C]MC1 successfully imaged upregulated COX-2 in the arthritic hand and shoulder and apparently in the brain. Uptake was blocked by celecoxib, a COX-2 preferential inhibitor.

**Conclusions:**

Taken together, these results indicate that [^11^C]MC1 can image and quantify COX-2 upregulation in both monkey brain after LPS-induced neuroinflammation and in human peripheral tissue with inflammation.

**Trial registration:**

ClinicalTrials.gov NCT03912428. Registered April 11, 2019.

## Background

Neuroinflammation is a complex and dynamic pathophysiological process that occurs in response to trauma, infection, and neuronal degeneration [1]. Positron emission tomography (PET) is an in vivo method to detect biochemical changes with high sensitivity and can serve as a useful tool for studying neuroinflammation. Nevertheless, PET imaging of neuroinflammation has largely been restricted to studies of either glucose metabolism (using [^18^F]2-fluoro-2-deoxy-d-glucose (FDG)) or translocator protein 18 kDa (TSPO), which is highly expressed in activated microglia and reactive astrocytes (for a review, see [2]). However, both glucose metabolism and TSPO have limitations as neuroinflammatory markers. For example, glucose metabolism is modulated by many non-inflammatory factors, including neuronal activity, and TSPO is expressed in non-inflammatory cells (e.g., vascular endothelium) [3]. In addition, TSPO slowly increases after brain injury and takes several days to reach maximal density, which is maintained for weeks after the apparent resolution of the inflammation [4, 5].

The cyclooxygenase (COX) isozymes, COX-1 and COX-2, catalyze the rate-limiting step in the synthesis of several inflammatory prostanoids and are inhibited by nonsteroidal anti-inflammatory drugs (NSAIDs) such as aspirin and ibuprofen. COX-1 is a constitutively expressed “housekeeping” enzyme present in almost all tissues and is not induced by inflammation. In contrast, COX-2 is the product of an immediate-early gene that is quickly and dramatically upregulated by inflammation, both at the transcript and protein levels [6, 7]. This rapid increase over a few hours and its similarly rapid return to baseline indicate that COX-2 is better suited for reflecting rapid changes in inflammatory response than TSPO.

To date, the development of PET radioligands for COX-2 has had limited success. Prior radiolabeled analogs of COX-2 inhibitors except for one [8] provided little specific binding, did not control for delivery of radioligand to the brain [9], or were only tested in cell culture [10, 11]. Our laboratory recently developed two novel COX PET tracers—[^11^C]PS13 for COX-1 and [^11^C]MC1 for COX-2—with 1 nM potency and 1000-fold selectivity for their cognate isozymes [12–14]. Using [^11^C]PS13, a recent study from our laboratory showed that COX-1 is constitutively expressed in monkey brain at baseline in healthy animals [15]. In contrast, using [^11^C]MC1, no specific (i.e., displaceable) binding to COX-2 in brain at baseline was observed; rather, specific binding was seen only in the ovary [15].

The present study sought to determine whether [^11^C]MC1 could image COX-2 in both monkey brain and human peripheral tissue during inflammation. In monkeys, the study assessed the ability of [^11^C]MC1 to image and quantify COX-2 after its upregulation by either one or two intracerebral injections of lipopolysaccharide (LPS), a potent inflammogen. As a comparator, COX-1 was measured using [^11^C]PS13. To verify and extend our PET results, the postmortem brains of these monkeys were histologically examined to identify the density and localization of COX-1 and COX-2 at the gene transcript or protein level. To determine whether [^11^C]MC1 could detect COX-2 in human organs, we imaged individuals with rheumatoid arthritis, given that COX-2 is markedly elevated in the affected joints of this autoimmune disease [16].

## Methods

### Study design

Both human and monkey studies were exploratory and lacked any prior results to calculate sample size or establish rules for ceasing data collection.

### Surgical procedures and LPS stereotaxic injections in monkeys

All procedures involving animals were approved by the National Institute of Mental Health Animal Care and Use Committee and complied with the Institute of Medicine Guide for the Care and Use of Laboratory Animals.

Surgical procedures were performed in a veterinary operating facility under aseptic conditions. Vital signs were monitored throughout the procedure. A pre-operative MRI determined the stereotaxic coordinates for the site of the LPS injection in the right putamen. The skull region above the target site was exposed by retracting the skin, fascia, and muscle in anatomical layers. A small region of cranial tissue was then removed (~ 1 cm diameter) to access the dura mater, into which an incision was made to provide access for the infusion apparatus.

LPS from *Escherichia coli* O26:B6 (Sigma-Aldrich, St. Louis, MO) was dissolved in preservative-free 0.9% sodium chloride (APP Pharmaceuticals, Los Angeles, CA) at a concentration of 1 μg/μL under sterile conditions. This solution was then loaded into a 25-μL glass syringe with a 31-gauge needle (Hamilton Co., Franklin, MA) and mounted in a Nanomite pump (Harvard Apparatus, Cambridge, MA). The needle was lowered through the incision in the dura mater to the pre-calculated target site in the right putamen, and LPS (10 μg) was infused at a rate of 0.5 μL/min over 20 min. After infusion, the needle was left in situ for 10 min to allow pressure from the infusate to dissipate. The needle was then slowly removed, and the soft tissues were sutured together in anatomical layers.

### Human participants

Two female participants diagnosed with rheumatoid arthritis (ages 44 and 46) and two healthy controls (one 73-year-old man and one 66-year-old woman) participated in the study. The diagnosis of rheumatoid arthritis was based on published criteria [17]. The two control participants were medically and psychiatrically healthy based on medical history, physical examination, blood and urine laboratory testing, and electrocardiogram. None of the four participants had taken any kind of NSAID for 2 weeks nor aspirin for 4 weeks prior to the PET scans. TSPO affinity type was determined by genetic analysis as previously described [18], and all four participants were found to be high-affinity binders. Three of the four participants underwent both [^11^C]MC1 and [^11^C]ER176 scans and one of the two control participants only underwent the [^11^C]MC1 scan. The interval between the PET scans with two different radioligands was 3 to 10 days. Two scans for each radioligand were obtained on the same day, with the first serving as the baseline scan and the second as a blocking study using 400 mg of the COX-2 preferential inhibitor celecoxib, which was administered orally 2–2.5 h before the second injection of the radioligand. Written informed consent was obtained from all participants, in accordance with the National Institutes of Health (NIH) Institutional Review Board (Protocol 19-M-0079 and NCT03912428).

### Monkey PET procedures

[^11^C]PS13 [14], [^11^C]MC1 [12], and [^11^C]PBR28 [19] were prepared as previously described, with high molar activity at the time of injection (Table S2 in Additional file 1).

The monkeys were initially immobilized with ketamine hydrochloride (10 mg/kg, i.m.) and subsequently anesthetized with 1.0–3.0% isoflurane and 98% O_2_. After injection of the radioligand (185–370 MBq), scans were acquired for 90 or 120 min using a Focus 220 PET camera (Siemens Medical Solutions, Knoxville, TN), and the images were reconstructed with Fourier rebinning and filtered backprojection. During the scan, arterial blood sampling was performed to obtain the radiometabolite-corrected input function for quantification. Because we were limited by the total blood volume that could be drawn per day from a monkey, some studies had reduced or no arterial samples. In those situations, standardized uptake value (SUV) from 60–90 min was used for cross-comparison instead. No arterial blood samples were obtained for the [^11^C]PBR28 scans. Electrocardiogram, body temperature, heart rate, and respiratory rate measures were monitored throughout the scan.

To normalize brain uptake, the concentration of parent radioligand was measured in plasma as previously described [20]. Whole blood samples (0.5 mL each) were drawn from the femoral artery at 15-s intervals for the first 120 s followed by 1–4 mL samples at 3, 5, 10, 30, 60, 90, and 120 min. In all studies, plasma free fraction was measured by ultrafiltration [21].

### Human PET procedures

[^11^C]MC1 [12] and [^11^C]ER176 [22, 23] were prepared as previously described under our Investigational New Drug Applications 142,872 and 122,236, respectively. Molar activity at the time of injection was 57 ± 26 GBq/μmol for [^11^C]MC1 and 69 ± 18 GBq/μmol for [^11^C]ER176, respectively.

Whole-body PET scans were obtained on a Biograph mCT (Siemens Healthineers; Erlangen, Germany) scanner in three dimensions; transmission data for attenuation correction were acquired in two dimensions before radioligand injection. Following a 1-min intravenous bolus injection of [^11^C]MC1 (544 ± 29 MBq) or [^11^C]ER176 (547 ± 17 MBq) and a 1-min interval, whole-body dynamic emission scans in seven to eight different bed positions were obtained from head to upper thigh for 90 min in 12–13 frames. The injected mass dose was 0.19 ± 0.10 nmol/kg for [^11^C]MC1 and 0.13 ± 0.04 nmol/kg for [^11^C]ER176, respectively. PET images were reconstructed with ordered subset expectation maximization with time of flight and resolution recovery.

### Brain image processing and kinetic analysis in monkeys

Three steps were followed to calculate distribution volume (*V*_T_) as a measure of enzyme density in monkey brain. First, dynamic PET images were co-registered to MRI templates [13]. Second, time-activity curves were generated using pre-defined regions of interest, including putamen, caudate, prefrontal cortex, and cerebellum in this template space. Third, to obtain *V*_T_, the Logan graphical approach was performed using regional time-activity curves and the serial concentrations of parent radioligand in arterial plasma (PMOD version 3.7, PMOD Technologies Ltd.; Zurich, Switzerland).

### Brain tissue preparation for histology

After completing the PET studies, monkeys were anesthetized with 10 mg/kg ketamine and 1 mg/kg xylazine, euthanized with sodium pentobarbital (25–30 mg/kg, i.v.), and then transcardially perfused with 0.9% (w/v) saline.

The entire brain was sliced into 5-mm-thick coronal blocks and subsequently divided into six pieces that were embedded into the optimum cutting temperature compound. Snap frozen blocks were made by cooling in dry ice methylbutane (Fisher Chemical, Fairlawn, NJ). Serial 10 μm cryo-sections of the brain were cut and mounted on Plus charged slides for histology and immunofluorescence. Coronal brain sections (10 μm thick) were placed on Superfrost Plus slides (Fisher Scientific, Pittsburgh, PA) and dried at − 20 °C for fluorescent multiplex in situ hybridization (FISH).

### Histology and immunofluorescence

Hematoxylin and eosin (H&E) stains were used to evaluate brain morphology. Digital histology images were obtained by scanning whole histology slides using a Nanozoomer digital slide scanner DNP RS 2.0 (Hamamatsu, Honshu, Japan).

For immunohistochemistry, the frozen slides were fixed with 4% paraformaldehyde for 5 min and incubated with 10% normal goat serum in PBS for 30 min to block non-specific binding. A mixture of primary antibodies for COX-2 (rabbit polyclonal, ab15191; Abcam, Boston, MA) and either CD68 (mouse monoclonal, ab955; Abcam) or neutrophil elastase (rabbit polyclonal, ab21595; Abcam) were applied to the slides, which were then incubated in a moisture chamber at 4 °C overnight. Slides were washed three times with PBS then incubated with Alexa Fluor 546 conjugated goat anti-mouse immunoglobulin G and Alexa Fluor 633 goat anti-rabbit immunoglobulin G for 1 h at room temperature. The slides were then counter-stained with Hoechst DAPI dye for 15 min and mounted with Fluoromount G (EM Sciences, Hatfield, PA). The slides were imaged using a Zeiss 780 confocal microscope with a × 20 objective, and the images were subsequently analyzed using ImageJ software (http://rsb.info.nih.gov/ij/).

### Fluorescent multiplex in situ hybridization

FISH was performed according to the manufacturer’s instructions using RNAscope fluorescent multiplex assay for fresh-frozen tissue (Advanced Cell Diagnostics (ACD)/Bio-Techne, Newark, CA). Slides were fixed for 15 min at 4 °C in 10% neutral-buffered formalin. Sections were then dehydrated and digested with protease III (all reagents from ACD) for 30 min at room temperature. Sections were incubated for 2 h at 40 °C in a hybridization oven (HybEZ) with ACD probes for *Macaca mulatta Cox2* and neuron-specific enolase (*Eno2*). Sections were then incubated with preamplifiers, amplifiers, and fluorescent dyes assigned to specific probes: Cy3 for *Cox2* and Cy5 for *Eno2*. As an internal control, adjacent sections were also stained with 3-plex positive control probes for the housekeeping genes RNA Polymerase II Subunit A (*Polr2a*), Cyclophilin B (*Ppib*), and ubiquitin C (*Ubc*). After counterstaining with 1:10,000 DAPI (Life Technologies, Grand Island, NY) for 1 min, slides were cover-slipped with polyvinyl alcohol mounting medium with DABCO (1,4-diazabicyclo[2.2.2]octane, Sigma, St. Louis, MO) to prevent fading.

Expression levels of *Cox2* and *Eno2* mRNA transcripts were quantified in putamen and cortex. At least ten images per area were used for quantification, containing > 2000 cells. All image analysis steps were performed using Volocity 6.3 (PerkinElmer, Shelton, CT). The analysis was carried out in three main steps: nuclear segmentation, FISH spot identification, and cell classification/scoring. First, individual nuclei were segmented using the DAPI channel. To correct for under-segmentation caused by cell clustering, large clusters of nuclei were first identified and then processed through a shape-based watershed to divide individual nuclei. DAPI masks greater than 20 μm^2^ were excluded, as were all edge objects. Individual masks were then expanded by 2 μm from the edge to include peripheral labeling. The resulting region of interest for each cell nucleus was used as a search region for spot counting in the Cy3 and Cy5 channels. A threshold (two standard deviations higher than the median value) was then applied to each channel to detect bright spots in the DAPI mask. These were then further filtered based on size criteria to prevent false detection caused by background noise. Spots that overlapped spatially across two channels were counted as auto fluorescence and excluded from the analysis. Individual cells were counted as expressing *Cox2* or *Eno2* mRNA if two or more fluorescent dots were present in the DAPI mask. All acquired single-spot level data were exported as text files and further analyzed in GraphPad Prism 5 and Microsoft Excel 2015.

### Quantitative ELISA measurement of expressed COX-2 protein

Monkey prostaglandin endoperoxide synthase 2 (PTGS2) ELISA kit (MyBiosource, San Diego, CA) was used to quantify the absolute concomitant concentration of COX-2. Methods were performed as per the manufacturer’s recommendations. Briefly, standard, control, and homogenate samples were added into 96-well strip plates. Horseradish peroxidase conjugate was then added, and the plate was incubated for 1 h at 37 °C. The plate was washed six times with wash solution and incubated with chromogens A and B at 500 rpm for 15 min at 37 °C. Stop solution was added and absorbance at 450 nm was read in a SpectraMax i3 microplate reader (Molecular Devices, San Jose, CA).

Standard curves were fitted using the corrected absorbance of known concentrations of COX-2, and the samples were interpolated within the curve to yield the COX concentrations in ng/mL of homogenate using GraphPad Prism.

### Statistical analysis

For each of the COX isozyme FISH assays, a non-parametric one-way analysis of variance (ANOVA) was used to determine a possible region-by-injection effect. Newman-Kuel tests were used to evaluate regional differences, and *t* tests were used to compare LPS-injected and non-treated animals. Bonferroni post-hoc tests were used to evaluate omnibus main effects. All analyses were run using GraphPad Prism 5.0 (https://www.graphpad.com/scientific-software/prism/).

## Results

### Monkey experimental timeline

Four male rhesus monkeys (*Macaca mulatta*) were scanned with [^11^C]MC1 for COX-2, [^11^C]PS13 for COX-1, and/or [^11^C]PBR28 for TSPO, before and after injection of LPS in the right putamen (Fig. S1 in Additional file 1). Monkeys 1 and 2 had one LPS injection; monkeys 3 and 4 had a second injection 33 and 44 days, respectively, after the first injection, again into the right putamen. The purpose of the studies in each animal differed slightly. For monkey 1, we sought to measure the effect of a single LPS injection on COX-2. For monkey 2, we sought to measure the time course of COX-2 expression after a single LPS injection. For monkeys 3 and 4, we sought to measure the effect of a single LPS injection on COX-1 and the effect of two LPS injections on COX-2.

### MRI and TSPO PET in monkeys

**Fig. 1:**
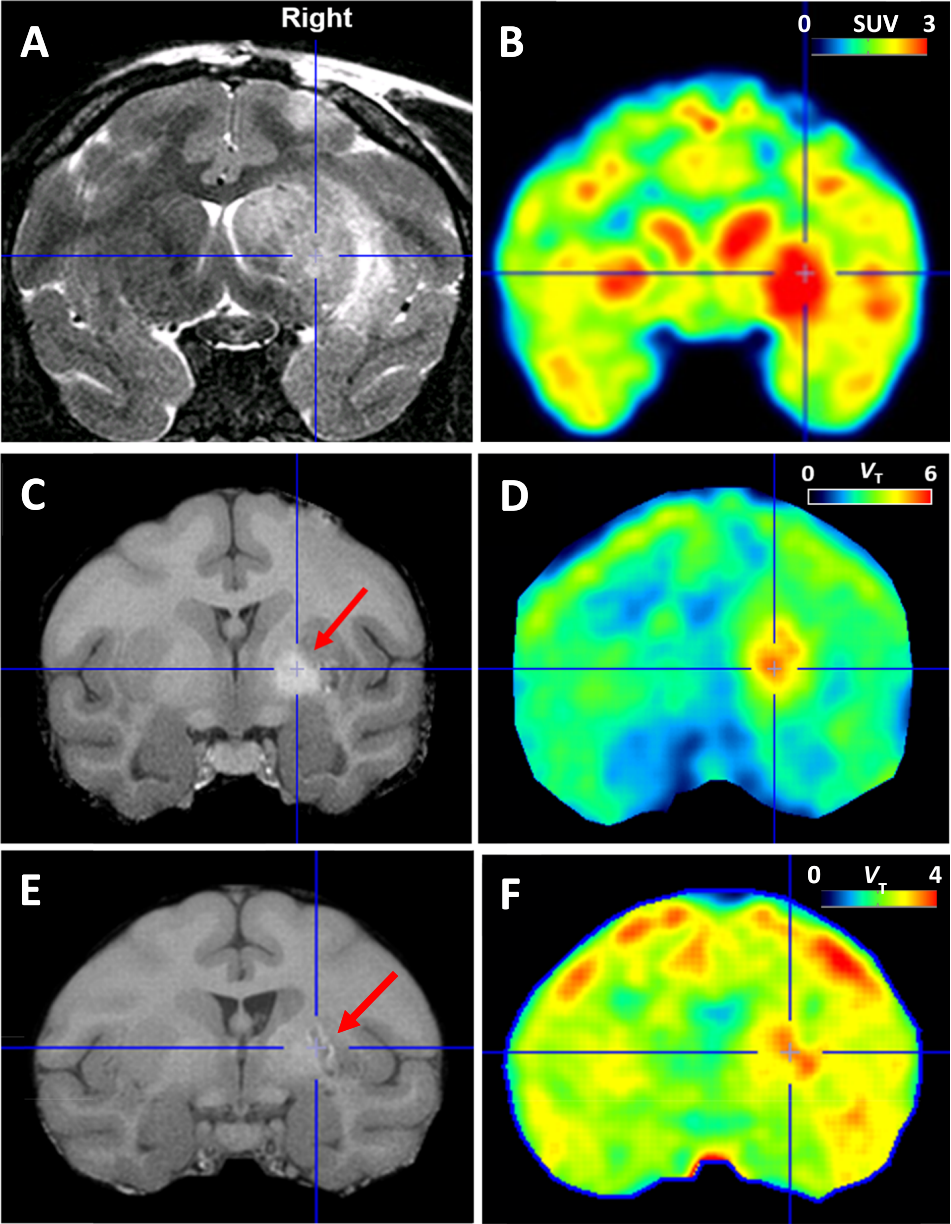
Edema and/or hemorrhage after the first and second injections of lipopolysaccharide (LPS). **a, b** After the first injection of LPS in monkey 2, the T2 magnetic resonance images (MRI) (**a**) showed edema surrounding the injection site in the right putamen and a leftward shift of the midline. The [^11^C]PBR28 scans (**b**) obtained on post-LPS day 3 showed increased TSPO binding in the right putamen. The orthogonal cross-hairs mark the injection site in the right putamen. **c, d** MRI and COX-2 scans in monkey 3. The T1-weighted MRI (**c**) was obtained after the first LPS injection but prior to the second one and showed an intracerebral hemorrhage, which is localized with cross-hairs. This hemorrhage was adjacent to, but overlapped with, the injection site, marked with a red arrow. The COX-2 scan (**d**) in monkey 3 showed high uptake overlying the pre-existing hemorrrhage and not the injection site. **e, f** MRI and COX-2 scans in monkey 4 showed results similar to those in monkey 3. The T1-weighted MRI (**e**) obtained between the two LPS injections showed an intracerebral hemorrhage that was offset from the injection site. The COX-2 scan (**f**) showed high uptake overlying the pre-existing hemorrhage and not the injection site

Structural MRI and TSPO PET scans were used as positive controls to confirm robust neuroinflammation after a single LPS injection (Fig. 1). T2-weighted MRI scans in both monkey 1 and monkey 2 showed structural deformations in the ipsilateral putamen and caudate, marked edema at the injection site, and a pronounced leftward shift of the brain midline (Fig. 1a). In addition, TSPO binding in monkey 2 measured with [^11^C]PBR28 (no blood sampling) was increased in the right putamen on post-LPS day 3 compared to day 1 (Fig. 1b). That is, the ratio of uptake between the right and left putamen increased from 1.19 on day 1 to 1.30 on day 3, consistent with the relatively slow time-course of TSPO expression following brain injury [24].

### COX-2 at baseline and after first LPS injection in monkeys

**Fig. 2:**
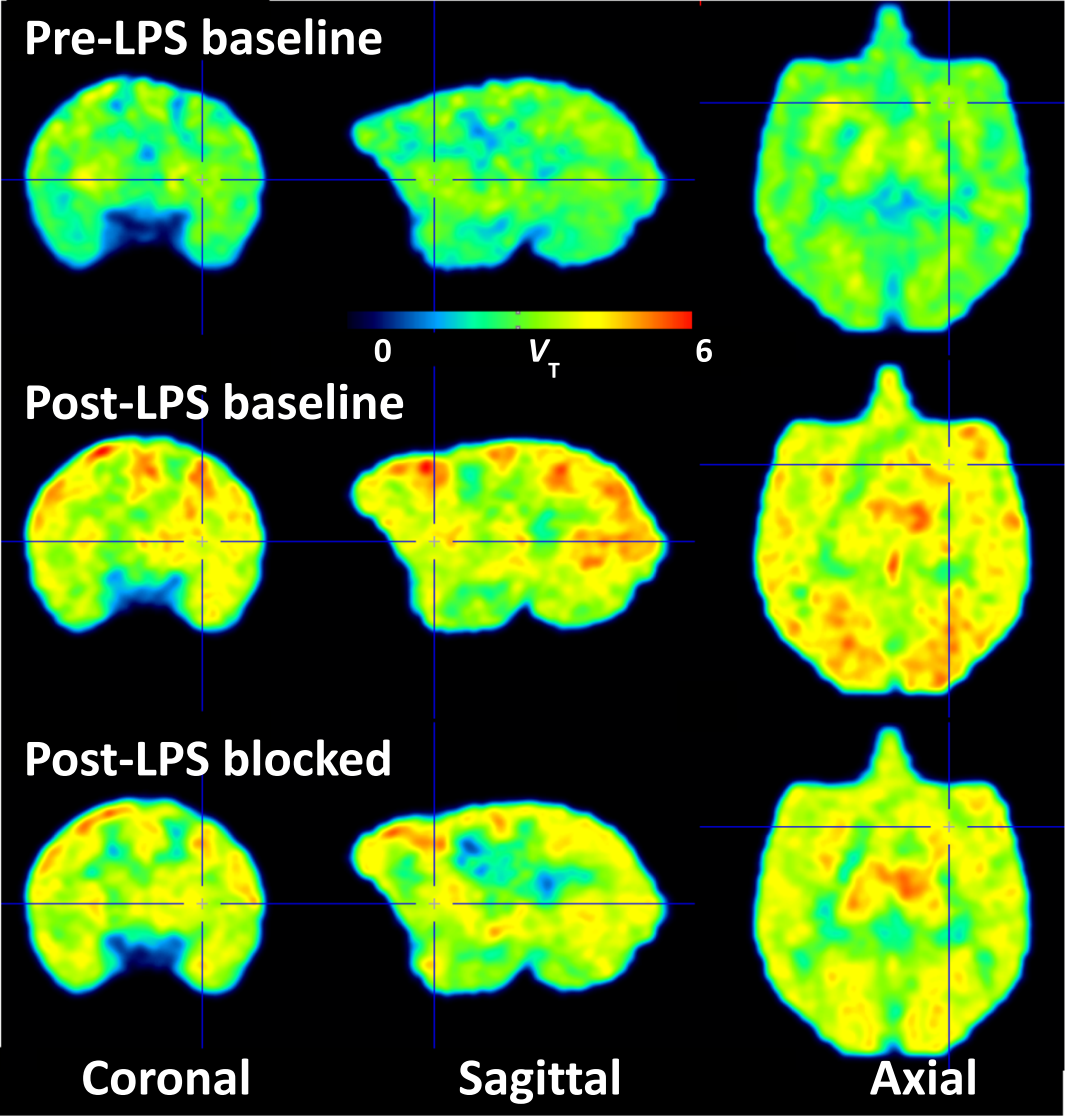
Parametric images of distribution volume (*V*_T_) showing [^11^C]MC1 uptake before (top row) and on day 1 (middle row) after the first lipopolysaccharide (LPS) injection in monkey 2, followed by pharmacological blockade by MC1 (0.3 mg/kg, i.v.) on the same day (bottom row). LPS increased [^11^C]MC1 uptake globally, which was blocked by cold parent. Orthogonal cross-hairs show the injection site, right putamen. The blockade by MC1 was incomplete—i.e., 78% according to the Lassen plot (Fig. 4). Thus, higher doses of MC1 would have caused even greater blockade

**Fig. 3:**
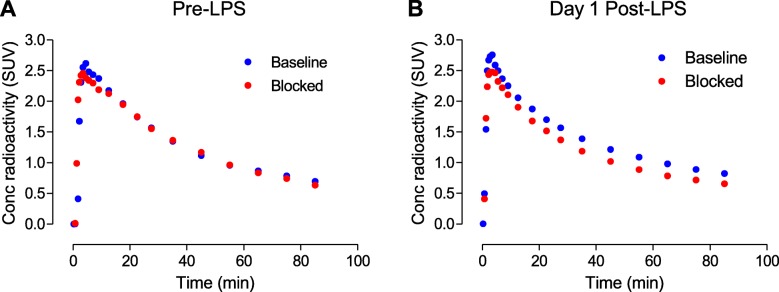
Whole brain time-activity curves of [^11^C]MC1 uptake before (**a**) and on day 1 after the first lipopolysaccharide (LPS) injection (**b**) in monkey 2, under baseline and blocked conditions by MC1 (0.3 mg/kg, i.v.) on the same day. LPS increased whole brain [^11^C]MC1 uptake, which was blocked by cold parent

Before LPS injection, no specific binding to COX-2 was detected using [^11^C]MC1 (Fig. 2, top row, and Fig. 3a). Non-radioactive MC1 (0.3–1 mg/kg, i.v.), which has high potency for COX-2 (*IC*_50_ = 1 nM) and 1000-fold selectivity over binding to COX-1 (*IC*_50_ > 1000), was used as the blocking agent [13] (Fig. 3a). *V*_T_ values of [^11^C]MC1 in whole brain were similar before and after blockade with MC1 (3.63 ± 0.19 vs. 3.45 ± 0.46 mL cm^−3^, *p* = 0.58, *n* = 4 animals).

A single LPS injection (10 μg) into the right putamen globally increased [^11^C]MC1 uptake in the brain (Fig. 2, middle row, and Fig. 3b). For the two animals with arterial input measurements (monkeys 1 and 2), whole brain *V*_T_ increased by 32% in monkey 1 (from 3.47 to 4.59 mL cm^−3^) and by 42% (from 3.76 to 5.32 mL cm^−3^) in monkey 2. In confirmation that the increased radioactivity was specifically associated with COX-2, pharmacological blockade with non-radioactive MC1 (0.3 mg/kg, i.v.) decreased whole brain *V*_T_ by 21% (from 4.59 to 3.62 mL cm^−3^) in monkey 1 and by 16% (from 5.32 to 4.45 mL cm^−3^) in monkey 2 (Fig. 2, bottom row, and Fig. 3b).

**Fig. 4:**
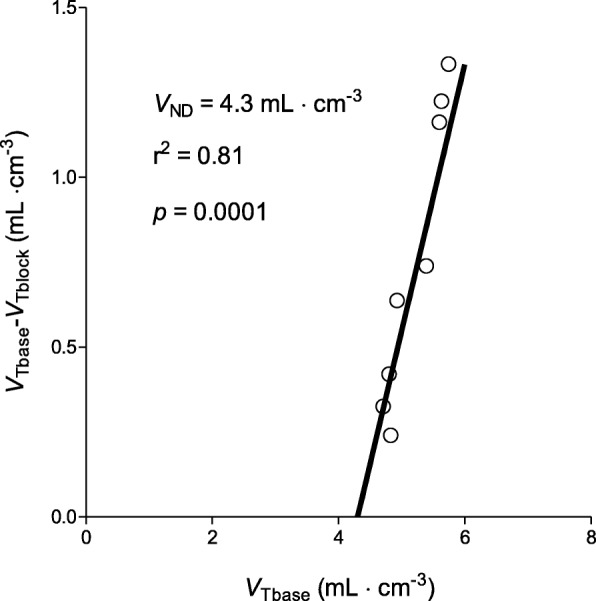
Lassen plot to determine cyclooxygenase-2 (COX-2) occupancy and non-displaceable uptake (*V*_ND_) of [^11^C]MC1 in brain after a single lipopolysaccharide (LPS) injection in monkey 2. Each point represents a brain region from a single animal scanned twice in one day: at baseline and after enzyme blockade by MC1 (0.3 mg/kg i.v. administered five minutes before the second radioligand injection). The slope of the straight-line fit provides the occupancy (78%), meaning that MC1 occupied 78% of all COX-2 molecules. The x-intercept provides the *V*_ND_ (4.3 mL cm^−3^)

Specific binding in these two animals was also assessed using the Lassen plot, which analyzes multiple brain regions. In monkey 2, *V*_ND_ (nondisplaceable *V*_T_) was estimated to be 4.3 (*R*^2^ = 0.81, *p* = 0.0001) on day 1 post-LPS (Fig. 4). This resulted in a binding potential (*BP*_ND_) of 0.21. The Lassen plot for monkey 1 showed a data distribution that did not allow for reliable straight-line fitting. The cause of the non-linearity is unknown, but possibilities include regional variations in *V*_ND_ (which violate a requirement for the Lassen plot) as well as relatively low specific signal in this animal (which adds noise to the plot).

**Fig. 5:**
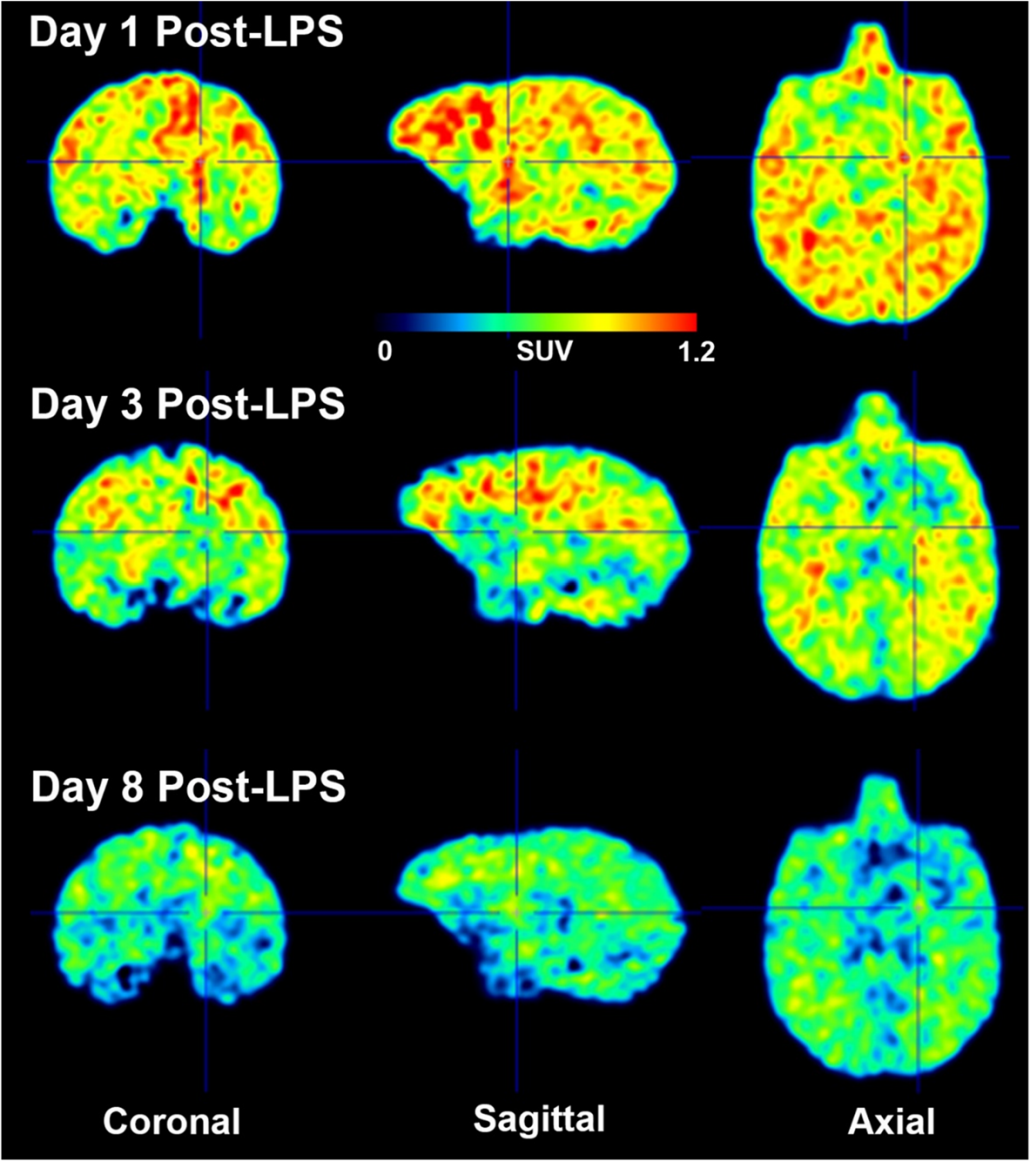
Time-course comparison of [^11^C]MC1 standard uptake value (SUV) images obtained on day 1, day 3, and day 8 after the first lipopolysaccharide (LPS) injection in monkey 2. Orthogonal cross-hairs show the injection site, right putamen. [^11^C]MC1 uptake was greatest on day 1, decreased on day 3, and was lowest on day 8

To measure the time-course of increased COX-2 after a single LPS injection, [^11^C]MC1 PET scans were performed in monkey 2 on days 1, 3, and 8 post-LPS (Fig. 5). Because of limits on the total amount of blood that could be collected, *V*_T_ could not be quantified in these scans. Instead, specific COX-2 expression was calculated as the difference of *SUV*_brain_*/SUV*_blood_ over 60–90 min between baseline and self-blocked conditions. This *SUV* ratio was greatest on day 1 (5.21), decreased on day 3 (1.37), and returned to almost pre-LPS levels on day 8 (1.04). On visual inspection, the effect appeared to be global, with no marked difference at the injection site relative to the whole brain.

**Fig. 6:**
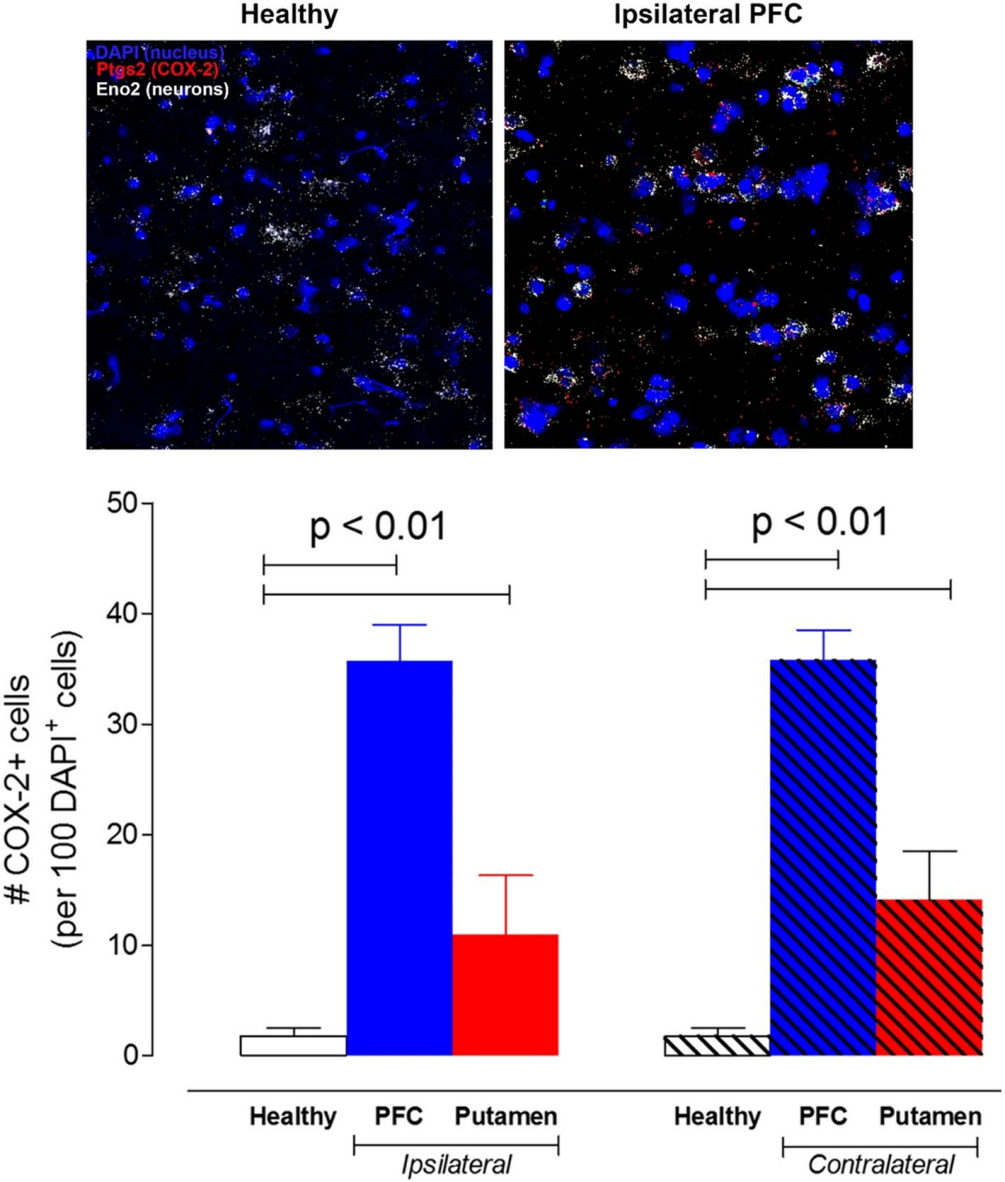
Quantitative fluorescent in situ hybridization (FISH) of the gene transcript of *PTGS2* (the gene that encodes COX-2) after a single lipopolysaccharide (LPS) injection in monkey 1. In contrast to healthy control, marked *PTGS2* transcript was detected in prefrontal cortex (PFC) and putamen, both ipsi- and contralaterally to the injection site in putamen. *PTGS2* transcript was primarily colocalized (~ 75%) in neurons in cortex and putamen

To confirm that LPS increased COX-2 expression, the density of the transcript for *PTGS2* (the gene that encodes COX-2) in postmortem brain was measured using FISH and the density of the expressed protein was quantified using ELISA. Monkey 1 was selected for these experiments because it was euthanized immediately after its COX-2 scan on day 1 post-LPS. *PTGS2* transcript was negligibly expressed in healthy brain tissue (i.e., pre-LPS) but increased after the first LPS injection by 6-fold in putamen and 20-fold in prefrontal cortex (Fig. 6). In comparison to the PET scans, these in situ hybridization measurements showed much greater increases in density of *PTGS2* transcript as well as an unexpected regional selectivity (prefrontal cortex > putamen).

To help address the apparent discrepancy between the PET and FISH results, COX-2 protein expression was also measured using quantitative ELISA in six brain regions of monkey 1, and these data were compared to data from the two control monkeys that were not injected with LPS (Table S1 in Additional file 1). The concentration of COX-2 (pmol/mg protein) in the six brain regions was 29% higher in monkey 1 (8.1 ± 1.5, range, 5.9–10.1) than in the two control animals (6.3 ± 1.2, range, 4.4–7.8, *p* = 0.027). Unlike the density of *PTGS2* transcript, the density of COX-2 protein increased uniformly in brain and by a similar amount to that observed with PET. That is, in monkey 1, protein expression increased by 29%, and *V*_T_ increased by 32% (from 3.47 to 4.59) when comparing pre-and post-LPS scans with [^11^C]MC1.

### COX-2 after second LPS injection

**Fig. 7:**
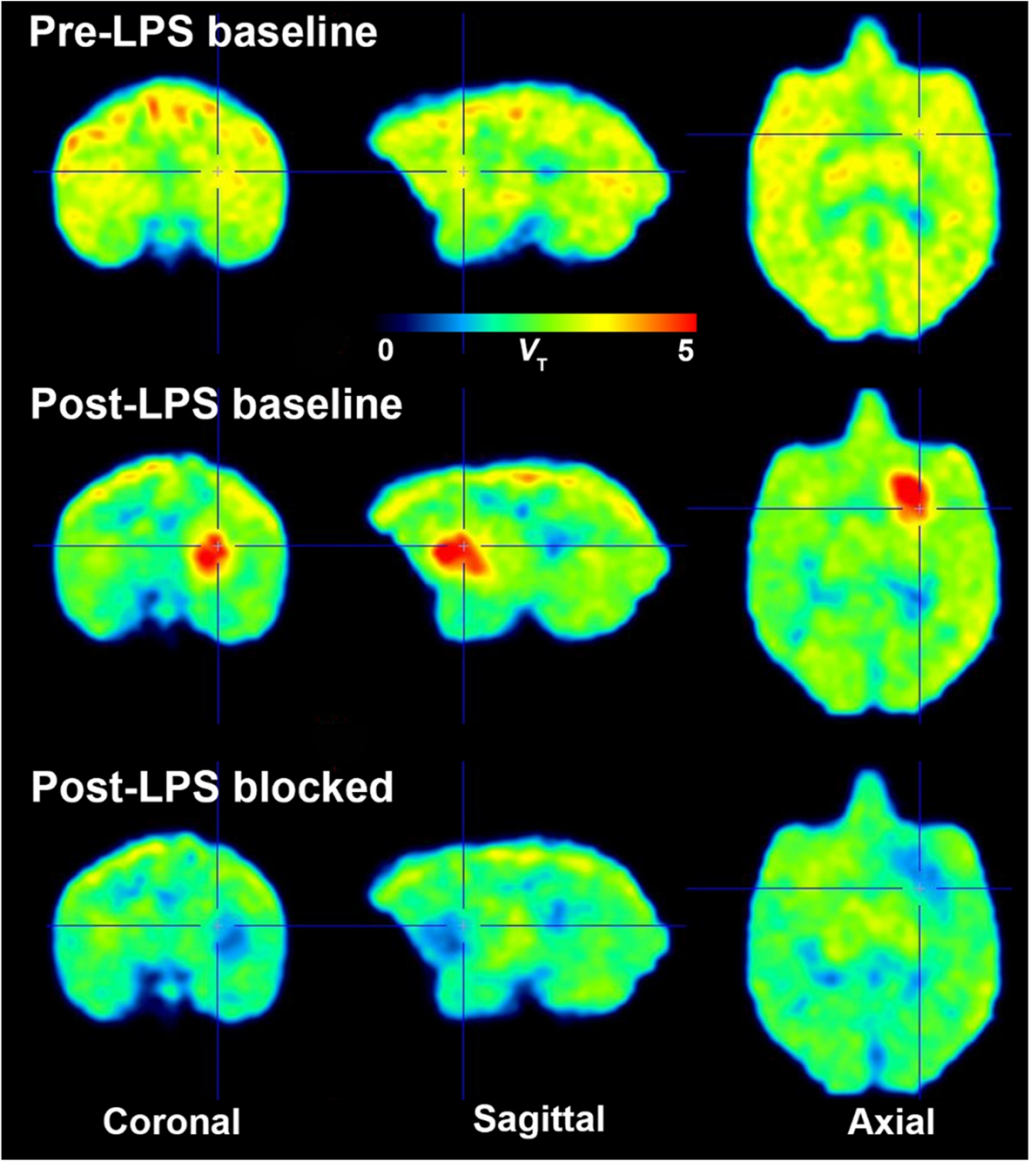
Parametric distribution volume (*V*_T_) images of [^11^C]MC1 uptake in monkey 3 before lipopolysaccharide (LPS) injection (top row), on day 1 after the second (LPS) injection (middle row), and after blockade by cold MC1 (1 mg/kg, i.v., bottom row). Orthogonal cross-hairs show the injection site, right putamen. [^11^C]MC1 uptake was markedly increased near the injection area in the right putamen after LPS injection. Cold MC1 blocked radioligand binding to cyclooxygenase-2 (COX-2) at the injection site to a level lower than that in the remainder of brain. Because this dose of MC1 achieved almost complete blockade (i.e., 75%), the residual uptake closely reflected nondisplaceable distribution volume (*V*_ND_). Thus, *V*_ND_ in the area of the lesion was reduced below that of normal brain

To examine whether a second LPS injection would cause an even larger global increase of COX-2, two animals (monkeys 3 and 4) were injected with LPS a second time after more than a month after the first LPS injection (Fig. S1 in Additional file 1). In monkey 3, a lesion adjacent to the injection site that had a marked increase of COX-2 binding and a marked decrease of nondisplaceable distribution volume (*V*_ND_) of the radioligand [^11^C]MC1 was unexpectedly identified (Fig. 7). On day 1 after the second LPS injection, *V*_T_ (mL cm^−3^) in these lesions near the right putamen increased from a pre-LPS value of 3.60 (mean of 3.71 and 3.48; individual values provided for monkeys 3 and 4, respectively) to 4.23 (mean of 4.99 and 3.46) (Fig. 7, middle row). Interestingly, MC1 blockade (1 mg/kg) reduced *V*_T_ to 1.61 (mean of 1.62 and 1.59) in the lesions and to 2.75 (mean of 2.61 and 2.88) in the remainder of the brain. This 2-fold lower residual activity in the lesions (1.61 vs. 2.75) was visible on the blocked scan (Fig. 7, bottom row, marked by red arrow) and meant that the lesions had lower *V*_ND_ than the remainder of the brain. If blocked *V*_T_ at the injection site was used as *V*_ND_, *BP*_ND_ for the lesions on day 1 after the second LPS injection was 1.77 (mean of 2.38 and 1.16). Using *V*_ND_ from Lassen plot, *BP*_ND_ for the remainder of the brain was 0.32 (mean of 0.39 and 0.24) and thus similar to that observed after the first LPS injection.

**Fig. 8:**
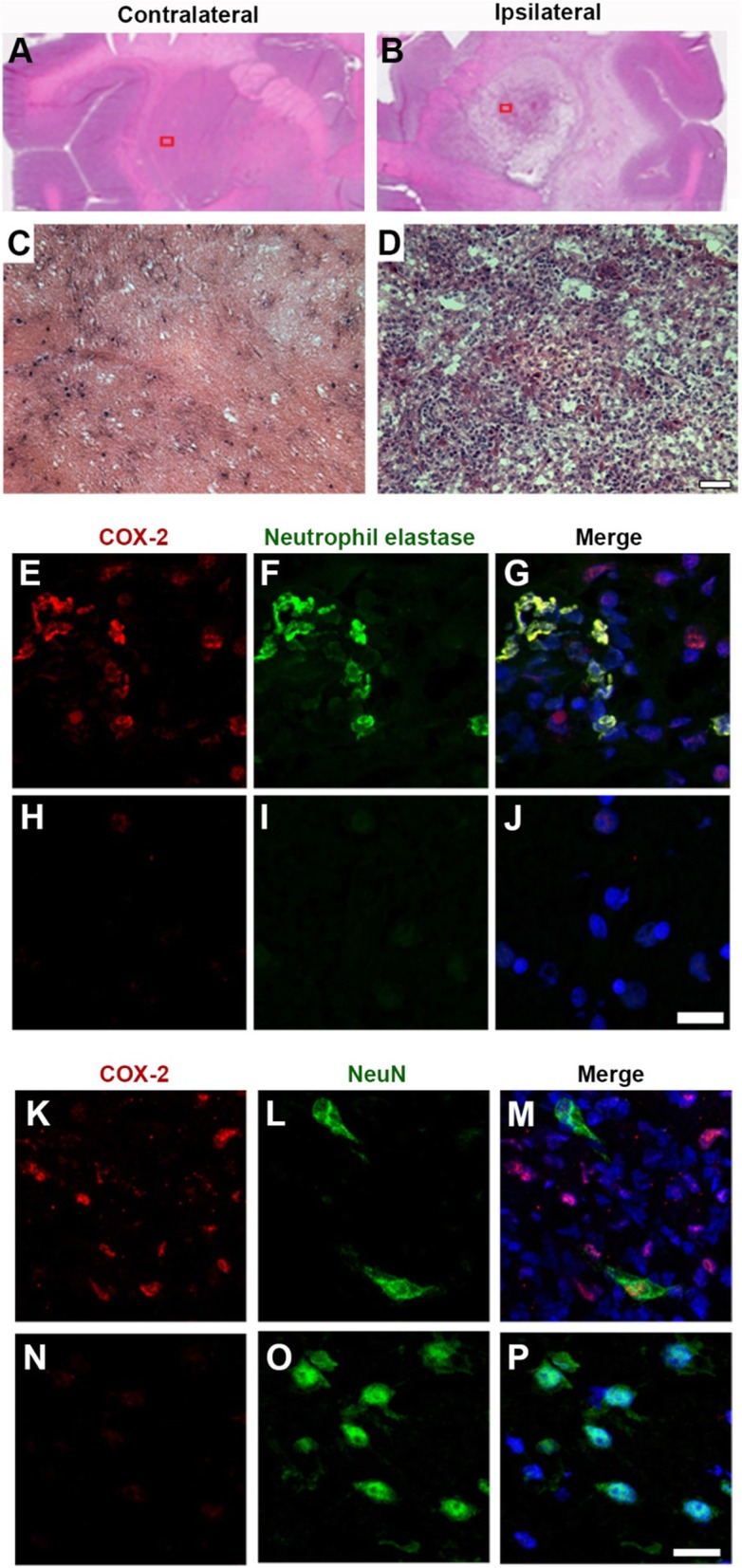
Postmortem histologic findings after the second lipopolysaccharide (LPS) injection in monkey 3. From **a** to **d** Cellular staining (hematoxylin and eosin (H&E)) of contralateral (**a**, **c**) and ipsilateral (**b**, **d**) putamen. The red square in **a** or **b** indicates the sampling area where each larger image (**c** or **d**) was taken. The ipsilateral putamen (**d**) showed a marked increase in the number of infiltrating leukocytes. The contralateral putamen (**c**) showed little or no change. Scale bar = 100 μm. From **e** to **j** Confocal photographs demonstrate the expression of cyclooxygenase-2 (COX-2) in neutrophils. The top panel (**e, f, g**) shows the expression of COX-2 (**e**) and neutrophil elastase (**f**). The merged fluorescent image (**g**) shows strong colocalization of COX-2 and neutrophil elastase. The contralateral hemisphere (**h, i**, j) shows negligible COX-2 (**h**) and neutrophil (**i**) staining. COX-2 (red), neutrophil elastase (green), nuclear protein (blue). Scale bar = 10 μm. From **k** to **p** Confocal photographs demonstrate the expression of COX-2 in neurons. The top panel (**k, l, m**) shows the expression of COX-2 (**k**) and neuronal nuclear protein (NeuN) (**l**) near the partially necrotic core. The merged fluorescent image (**m**) shows occasional co-localization of COX-2 in NeuN-positive cells. The contralateral hemisphere (**n, o,** p) showed only NeuN-positive cells. COX-2 (red), NeuN (green), nuclear protein (blue). Scale bar = 20 μm

In monkey 3, the lower nonspecific binding in the putamen after the second LPS injection reflected a spherical lesion about 0.5 cm in diameter. Postmortem histology showed a massive, but incomplete, loss of neurons and a dense infiltration of leukocytes, primarily neutrophils (Figs. 8a–j). A few COX-2-expressing neurons were also detected at the necrotic core and adjacent to it (Fig. 8k–p).

In monkeys 3 and 4, the increased uptake of [^11^C]MC1 likely resulted from a delayed effect from the first LPS injection rather than an acute effect of the second LPS injection. Both monkeys developed delayed intracerebral hemorrhage(s) after the first LPS injection, as seen on T1 MRI images obtained before the second LPS injection (Fig. 1c, e). These lesions were offset from the actual injection sites, and the increased uptake of [^11^C]MC1 overlaid the hemorrhage rather than the injection site. Given that intracerebral hemorrhage is a potent inflammogen, these results suggest that increased COX-2 positive binding would likely have been seen independent of a second LPS injection.

### COX-1 at baseline and after LPS

Unlike COX-2, COX-1 is constitutively expressed in brain, and LPS did not affect its density as measured with PET. Using PS13 (0.3 mg/kg i.v.) as the blocking agent, 49% of [^11^C]PS13 uptake in baseline brain (pre-LPS) was found to be specifically bound to COX-1, echoing our previous results [13]. A single LPS injection did not significantly alter *V*_T_ (mL cm^−3^) from a baseline value of 5.57 (mean of 6.39 in monkey 1 and 4.75 in monkey 2) to a post-LPS day 1 value of 5.11 (mean of 6.30 and 3.92).

### Incidental finding of TSPO and COX-2 binding associated with muscle hematoma

A TSPO-positive lesion in muscle outside the skull was incidentally observed in an animal that was being scanned for a separate study. A PET scan showed that uptake in the lesion was blocked by PK11195 (5 mg/kg i.v.), a selective TSPO ligand (Fig. S2 in Additional file 1). Palpation of the lesion found it to be soft and ovoid, about 13 × 10 × 10 mm. Biopsy of the tissue 2 weeks later showed a resolving hematoma, with dense collagenous connective tissue and neovascularization. Three weeks after the biopsy, the animal was scanned again with [^11^C]MC1 and the lesion was found to be negative for COX-2; instead, a new line of COX-2 positivity was present along the incision used for the biopsy. [^11^C]MC1 uptake was specifically bound to COX-2 as it was displaced by non-radioactive MC1 (1 mg/kg i.v.). Four additional weeks later (i.e., almost 9 weeks after the initial incidental PET scan), we scanned for TSPO again and found that the lesion was negative but that a clear line of TSPO positivity, blockable by PK11195, overlay the incision.

One interpretation is that the ovoid lesion was inflamed at the time of the first PET scan for TSPO and that the inflammation resolved by the time of biopsy and subsequent PET scans. The fact that the skin incision was positive for COX-2 3 weeks after the biopsy likely reflects the slow healing of this site as well as the fact that absorbable sutures had not yet been fully resorbed.

### COX-2 and TSPO imaging in participants with rheumatoid arthritis

**Fig. 9:**
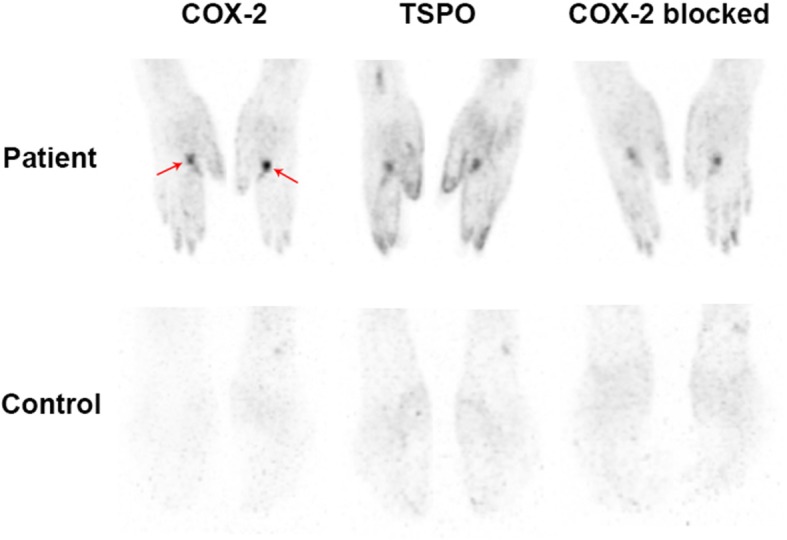
Human positron emission tomography (PET) images of cyclooxygenase-2 (COX-2) and translocator protein (TSPO) in patient 1 with rheumatoid arthritis and a healthy control. Increased [^11^C]MC1 uptake in the bilateral hand joints reflected increased COX-2 binding in Patient 1 compared to the healthy control. TSPO binding represented by [^11^C]ER176 uptake in the same individuals showed consistent distribution with COX-2 binding in the bilateral hand joints of patient 1. The increased [^11^C]MC1 uptake in patient 1 with rheumatoid arthritis was blocked by celecoxib

**Fig. 10.**
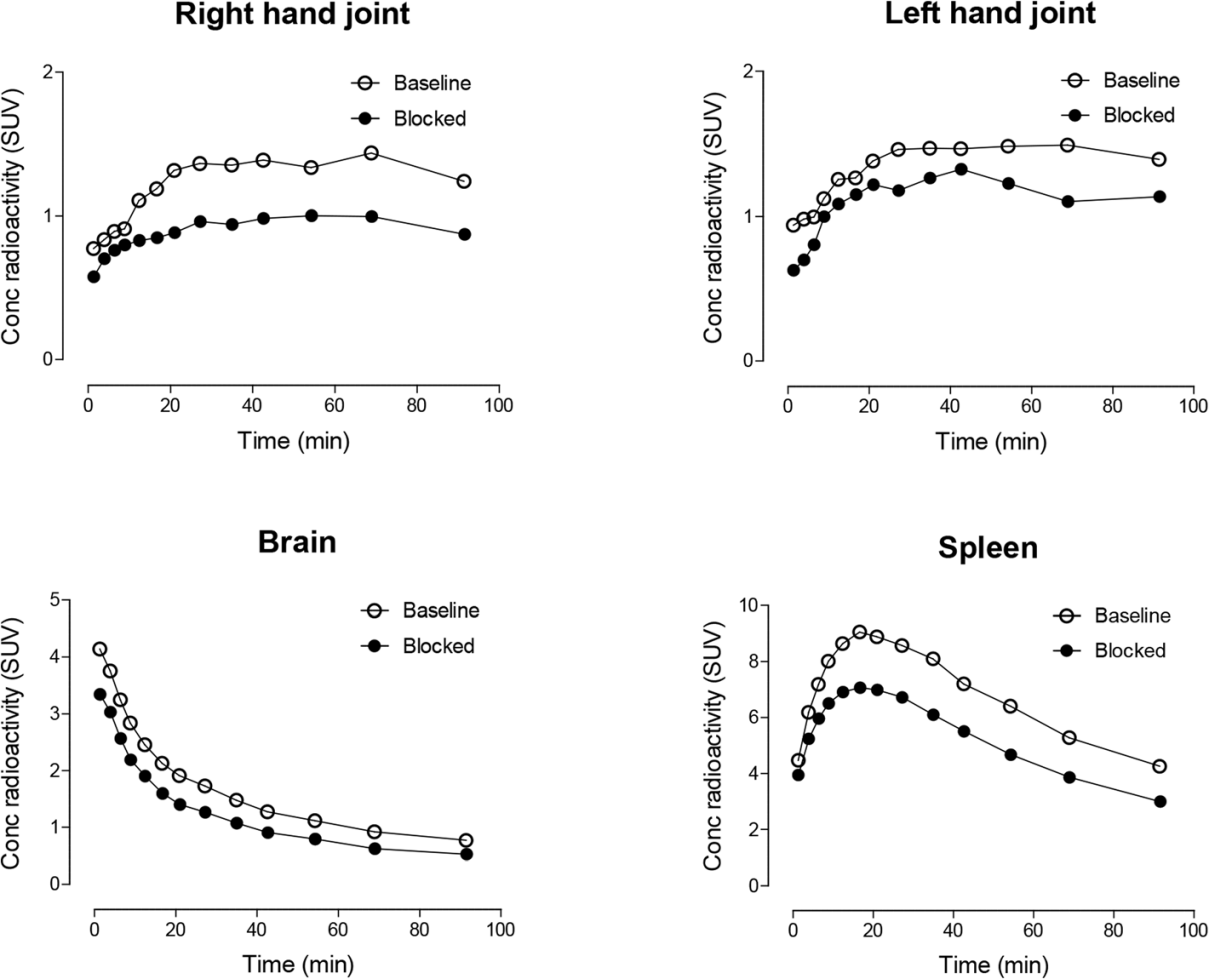
Time-activity curves of [^11^C]MC1 uptake in representative body parts of patient 1 with rheumatoid arthritis under baseline and blocked conditions. Celecoxib (400 mg) was used as the blocking agent

To assess whether [^11^C]MC1 can detect COX-2 in humans, we initially chose not to image the brain, given that it might be similar to healthy monkey brain, which shows no detectable binding. Instead, the joints of patients with rheumatoid arthritis were imaged because COX-2 is markedly elevated in the affected joints of this autoimmune disease [16]. Significantly higher uptake of [^11^C]MC1 (COX-2) and [^11^C]ER176 (TSPO) was observed in the hand, elbow, and/or shoulder joints of two individuals with rheumatoid arthritis compared to other joints in the same individuals as well as in the corresponding joints of two healthy controls (Fig. 9). These locations were consistent with the current symptoms of the individuals with rheumatoid arthritis; that is, patient 1 had pain and swelling in both hands, and patient 2 was affected in both shoulders, elbows, and hands. The specificity of [^11^C]MC1 uptake in these joints was assessed in a repeat scan acquired about two hours after administration of the COX-2 selective inhibitor celecoxib (400 mg PO). Celecoxib decreased uptake of [^11^C]MC1 in the affected joints (hands) by about 23% in patient 1 (Figs. 9 and 10), but minimally in patient 2 (data not shown), calculated as the concentration (SUV) of radioactivity from 0 to 90 min. As a negative control, the participants were also re-scanned with [^11^C]ER176 after celecoxib; as expected, this COX-2 inhibitor did not block uptake of the TSPO radioligand.

COX-2 imaging in these participants produced three unexpected findings. First, both individuals with rheumatoid arthritis had elevated uptake of [^11^C]MC1 in the brain, which was blocked 26% and 32% by celecoxib (Fig. 10). The distribution of radioactivity in brain was not uniform and had an order of neocortex > subcortical gray > cerebellum. This differential distribution allowed us to estimate specific and nondisplaceable uptake using the concentration of radioligand (SUV) in brain regions as a surrogate for fully quantified *V*_T_, which was corrected for the concentration of radioligand in arterial plasma. In the two individuals with rheumatoid arthritis, occupancy was estimated to be 56% and 60%, and the surrogate for *BP*_ND_ was 0.75 and 0.96 (Fig. S3 in Additional file 1). The two healthy participants had such small and variable displaceable uptake that the correlation was insignificant (*P* > 0.29). The simplified analysis using SUV rather than *V*_T_ is only valid if the two variables are well correlated, but we did not have arterial data to assess this key assumption. Second, one of the two individuals with rheumatoid arthritis had markedly increased uptake in the spleen, which was blocked 24% by celecoxib (Fig. 10). Third, the same individual with rheumatoid arthritis had marked uptake in the posterior lingual area, which was blocked 68% by celecoxib (data not shown). A subsequent MRI scan of the neck showed the area to be unremarkable.

## Discussion

This study found that [^11^C]MC1 is one of the first PET radioligands to successfully image and quantify COX-2, as demonstrated by an LPS model of neuroinflammation in nonhuman primates as well as a first-in-human study of participants with rheumatoid arthritis.

In four rhesus macaques before LPS injection, no specific (i.e., displaceable) uptake of [^11^C]MC1 was detected—i.e., *BP*_ND_ = 0. However, a single LPS injection into the right putamen globally increased COX-2 radioligand to a *BP*_ND_ of 0.3. Postmortem histology confirmed the widespread and uniform upregulation of expressed COX-2 protein. Notably, however, *PTGS2* transcript increased in a region-specific manner, with both right and left putamen showing two to three times more mRNA expression than the rest of the brain. Scanning after the second LPS injection in two monkeys showed unexpected lesions that were likely caused by intracerebral bleeding as a long-term effect of the first LPS injection. In monkey 3, the lesion was about 0.5 cm in diameter and had high uptake of the COX-2 radioligand and a *BP*_ND_ of 1.8. Postmortem analysis showed that the lesion had partial necrosis and heavy infiltration of leukocytes. COX-2 was primarily located in the leukocytes, which are known to upregulate COX-2 as part of the inflammatory response [25]. The lower *V*_ND_ in the lesion (Fig. 7) was likely caused by the loss of endogenous brain tissue, which has high lipid content and can store high concentrations of lipophilic drugs like [^11^C]MC1 (measured log*D* = 3.7) [26].

The specificity of [^11^C]MC1 uptake in monkey brain was confirmed in this study by blockade with the nonradioactive carrier MC1. A previous report noted that high uptake of [^11^C]MC1 in monkey ovaries could be blocked by celecoxib [15]. Thus, the specificity of [^11^C]MC1 uptake in monkey has now been demonstrated by blockade with two compounds that have different chemical structures.

Because leukocytes originate from the periphery, [^11^C]MC1 could presumably image COX-2 upregulation in peripheral inflammatory disorders. In support of this possibility, we incidentally found, in an unrelated monkey, a lesion in the skull muscle that was positive for TSPO and negative for COX-2 (Fig. S2 in Additional file 1). Biopsy showed the lesion to be a hematoma. Repeat scanning revealed that TSPO in the lesion itself resolved, but that a line of increased TSPO and COX-2 radioligand uptake appeared, located along the incision for the biopsy. In addition, as a control and comparison, COX-1 was imaged using the selective radioligand [^11^C]PS13 in monkey brain. In contrast to the COX-2-related findings, COX-1 was found to be present at baseline but not upregulated by a single LPS injection.

The utility of [^11^C]MC1 in imaging COX-2 upregulation in peripheral inflammatory disorders was confirmed by extending this study to two human participants with rheumatoid arthritis and two healthy controls. The actively inflamed joints of the individuals with rheumatoid arthritis showed both increased COX-2 and TSPO binding, possibly caused by increased number and activity of leukocytes in the synovial membranes [27]. The specificity of [^11^C]MC1 binding to COX-2 was further confirmed by a blocking study with celecoxib, a preferential COX-2 inhibitor; it should be noted, however, that the blocking effect was minimal in one participant with rheumatoid arthritis, perhaps due to limited bioavailability of the drug in response to a single dose or to the variable gastric absorption rate [28]. It remains unknown whether this single dose of celecoxib (400 mg) completely blocked radioligand binding in this preliminary study. Additional experiments with higher or repeated doses are required to clarify this issue.

### Inducible expression of COX-2 in monkey brain and in rheumatoid arthritis

The first LPS injection produced a localized increase of TSPO binding, as expected from prior studies in rodents [29]. We expected a similarly localized upregulation for COX-2, but instead found a widespread and relatively uniform increase throughout the brain (Fig. 2). While the cause of these global effects remains unknown, one possibility is that LPS leaked into systemic circulation, resulting in widespread infiltration in brain, as found in animals and humans following intravenously administered LPS [30].

These results demonstrate that COX-2 and TSPO reflect different aspects of the brain’s inflammatory response, including time-course and cellular localization. COX-2 can be upregulated and return to baseline within hours [31], whereas upregulation of TSPO takes days and can last for weeks [5, 24]. Under basal conditions, COX-2 is minimally present at low concentrations; after induction, it is largely located in neurons [32]. In contrast, TSPO is strongly expressed under basal conditions and upregulated primarily in microglia and astrocytes but minimally in neurons [33]. It should be noted that these differential responses might be advantageous when imaging neuroinflammation with radioligands for both TSPO and COX-2. For example—and with regard to time-course—prior TSPO studies primarily examined disorders marked by chronic neuroinflammation, such as Alzheimer’s disease. In fact, the slow time-course and long-lasting upregulation of TSPO appear well-suited to gathering time-weighted measures of long-lasting neuroinflammation. However, unlike TSPO, COX-2 might be able to measure short-lived inflammatory response as well as chronic inflammation. As an example, the rapid upregulation (2 to 4 h) of COX-2 after acute injury and its subsequent downregulation after removal of the stimulus or treatment with glucocorticoids [34] might be imaged with [^11^C]MC1.

The region-specific differences in COX-2 expression following the second LPS injection in monkeys suggests that [^11^C]MC1 may be effective for imaging peripheral inflammation for two reasons. First, our postmortem analysis showed that the localized signal derived largely from leukocytes, which could similarly be imaged in any organ of the body, assuming they are present at adequate density. Second, the lesion exhibited partial necrosis with loss of most endogenous brain cells, which have high fat content; indeed, 60% of the overall dry weight of the brain is fat [35]. The lower fat content in this region was reflected in lower *V*_ND_ (i.e., lower nonspecific uptake). Lower *V*_ND_, in turn, increased the ratio of specific to nondisplaceable uptake (i.e., *BP*_ND_), which is associated with greater sensitivity of measurement. Taken together, the evidence suggests that levels of COX-2 in healthy human and nonhuman primate brain are too low to be measured with [^11^C]MC1, but that LPS administration to monkeys, or peripheral inflammatory disorders in humans, drive COX-2 expression to measurable levels.

Because some organs, like joints and muscle, contain relatively low amounts of fat, [^11^C]MC1 could be used to image inflammation with high contrast and sensitivity in disorders such as arthritis and myositis. When extending this work by scanning two participants with rheumatoid arthritis and two healthy controls, we identified localized infiltration of leukocytes into the synovial membrane, a classic pathological finding in rheumatoid arthritis [36, 37] that has also been associated with high expression of COX-2 and TSPO [16, 27, 38, 39]. As expected, joints, which have lower fat content than that in the brain, demonstrated adequate measurement of COX-2 binding by [^11^C]MC1.

### Constitutive expression of COX-2 in monkey brain

Although results have been mixed, other studies identified COX-2 mRNA and/or protein in the postmortem brains of rodents and humans, showing that COX-2 is constitutively expressed, albeit at low levels [40, 41]. Similarly, in this study, quantitative ELISA measurements showed that COX-2 protein was present in the postmortem brain of two healthy monkeys (~ 3 pmol/mg protein). Based on these results, the limit of sensitivity of [^11^C]MC1 to detect COX-2 was identified as > 3 pmol/mg protein. It remains unknown whether the elevated COX-2 density reported in relative units, rather than absolute units, in several human disorders (e.g., Alzheimer’s disease [42]) is high enough to be measured with [^11^C]MC1. Efforts to identify and/or develop other ligands with greater specific binding (e.g., via higher affinity) and lower non-specific binding (e.g., via lower lipophilicity) are ongoing.

### Unexpected findings in human participants with rheumatoid arthritis

Three areas with unexpectedly elevated [^11^C]MC1 binding blocked by celecoxib were observed in human participants with rheumatoid arthritis: brain in both participants, and spleen and the posterior lingual area in one of the two participants. The brain appears to have had displaceable/specific binding to COX-2, which might reflect a central response to peripheral inflammation, as also suggested by a previous TSPO imaging study [43]; another possible explanation is the response of brain vasculature, immune cells associated with perivascular spaces, choroid plexus, or meninges [44]. The marked uptake in the spleen of one individual with rheumatoid arthritis presumably reflects COX-2 in leukocytes, but we have no explanation for why it was not found in the other participant. Lastly, the same participant with marked uptake in the spleen also had markedly high uptake in the posterior lingual area, with substantial blocking effect by celecoxib; this participant had no associated clinical symptoms nor showed abnormal findings on subsequent neck MRI. Clearly, additional experiments are needed to explain these intriguing preliminary results.

## Conclusions

This study, which used [^11^C]MC1 to examine COX-2 expression in both monkey brain and human peripheral tissue, demonstrated that [^11^C]MC1 is one of the first PET radioligands to successfully image and quantify COX-2 in vivo. The results suggest that [^11^C]MC1 could be a potentially powerful tool for assessing inflammatory response in the brain and periphery. Moreover, in tandem with a PET radioligand selective for COX-1, [^11^C]MC1 could be used to assess drug delivery and in vivo selectivity of NSAIDs used in therapeutic trials.

## Supplementary information

**Additional file 1.** (COX-2 Neuroinflammation J Neuroinflammation ADDITIONAL FILE1) (Microsoft Word format).

## Data Availability

The data that support the findings of this study are available from the corresponding author upon request. The data are not publicly available due to privacy or ethical restrictions.

## Abbreviations

ANOVA: Analysis of variance
*BP*_ND_: Binding potential
COX: Cyclooxygenase
DABCO: 1,4-Diazabicyclo[2.2.2]octane
*Eno2*: Neuron-specific enolase
FDG: [^18^F]2-Fluoro-2-deoxy-d-glucose
FISH: Fluorescent multiplex in situ hybridization
H&E: Hematoxylin and eosin
LPS: Lipopolysaccharide
NIH: National Institutes of Health
NSAID: nonsteroidal anti-inflammatory drug
PET: Positron emission tomography
*Polr2a*: RNA polymerase II subunit A
*Ppib*: Cyclophilin B
PTGS2: Prostaglandin endoperoxide synthase 2
SUV: Standardized uptake value
TSPO: Translocator protein 18kDa
*Ubc*: Ubiquitin C
*V*_ND_: Nondisplaceable distribution volume
*V*_T_: Distribution volume
