# Supplementary material for: PET measurement of cyclooxygenase-2 using a novel radioligand: upregulation in primate neuroinflammation and first-in-human study
Source: J Neuroinflammation. 2020 May 2;17:140. doi: 10.1186/s12974-020-01804-6 (PMC7195739; doi:10.1186/s12974-020-01804-6)
Supplement: Supplementary file 1 — Additional file 1. (COX-2 Neuroinflammation J Neuroinflammation ADDITIONAL FILE1) (Microsoft Word format). [file 12974_2020_1804_MOESM1_ESM.docx]

*Shrestha et al, “PET measurement of cyclooxygenase-2 using a novel radioligand: upregulation in primate neuroinflammation and first-in-human study”*

**Additional File1**

**Supplementary Table S1**. Concentration of cyclooxygenase-2 (COX-2) using quantitative ELISA in brain regions of Monkey 1 (euthanized on Day 9 post-lipopolysaccharide (LPS) injection) and two control monkeys^†^

|  | **Brain region** |  | **Conc. (fmol/mg protein)*** |  |
| --- | --- | --- | --- | --- |
| Monkey 1 | |  |  |  |
|  | Frontal cortex - ipsilateral |  | 8.5 |  |
|  | Frontal cortex - contralateral |  | 10.1 |  |
|  | Insula - ipsilateral |  | 9.3 |  |
|  | Insula - contralateral |  | 6.9 |  |
|  | Putamen -ipsilateral |  | 8.0 |  |
|  | Putamen - contralateral |  | 5.9 |  |
|  | Mean |  | 8.1 |  |
|  | SD |  | ±1.5 |  |
|  | *N* |  | 6 |  |
|  |  |  |  |  |
| Control 1 | |  |  |  |
|  | Right amygdala |  | 5.9 |  |
|  | Left amygdala |  | 4.5 |  |
|  | Hippocampus |  | 5.0 |  |
|  | Pre-frontal cortex |  | 7.0 |  |
|  | Occipital |  | 7.4 |  |
|  | Putamen |  | 6.6 |  |
|  |  |  |  |  |
| Control 2 | |  |  |  |
|  | Right amygdala |  | 6.1 |  |
|  | Left Amygdala |  | 5.6 |  |
|  | Hippocampus |  | 4.4 |  |
|  | Pre-frontal Cortex |  | 7.4 |  |
|  | Occipital |  | 7.8 |  |
|  | Putamen |  | 7.5 |  |
|  | Mean |  | 6.3 |  |
|  | SD |  | ±1.2 |  |
|  | *N* |  | 12 |  |
| * The concentration is the average of at least two measurements using ELISA | | | | |

^†^The two control monkeys were not part of the cohort of four studied monkeys and did not receive LPS injection

**Supplementary Table S2**. Three PET radioligands studied in four rhesus macaques before and after lipopolysaccharide (LPS) injection

|  | Pre-LPS  (mean ± SD) | Post-LPS  (mean ± SD) |
| --- | --- | --- |
| Rhesus monkeys (n=4) |  |  |
| Age (years) | 10.32 ± 2.35 | 11.56 ± 2.78 |
| Weight (kg) | 10.1 ± 1.5 | 10.3 ± 1.2 |
| [^11^C]MC1 | n=8 | n=12 |
| Injected activity (MBq) | 261 ± 30 | 302 ± 32 |
| Molar activity (GBq/µmol) | 113 ± 54 | 114 ± 41 |
| Injected mass (nmol/kg) | 0.30 ± 0.16 | 0.31 ± 0.09 |
| [^11^C]PS13 | n=4 | n=7 |
| Injected activity (MBq) | 209 ± 39 | 297 ± 14 |
| Molar activity (GBq/µmol) | 141 ± 94 | 137 ± 56 |
| Injected mass (nmol/kg) | 0.23 ± 0.19 | 0.21 ± 0.08 |
| [^11^C]PBR28 | n=2 | n=10 |
| Injected activity (MBq) | 252 ± 79 | 304 ± 73 |
| Molar activity (GBq/µmol) | 187 ± 74 | 183 ± 68 |
| Injected mass (nmol/kg) | 0.17 ± 0.10 | 0.21 ± 0.18 |

**
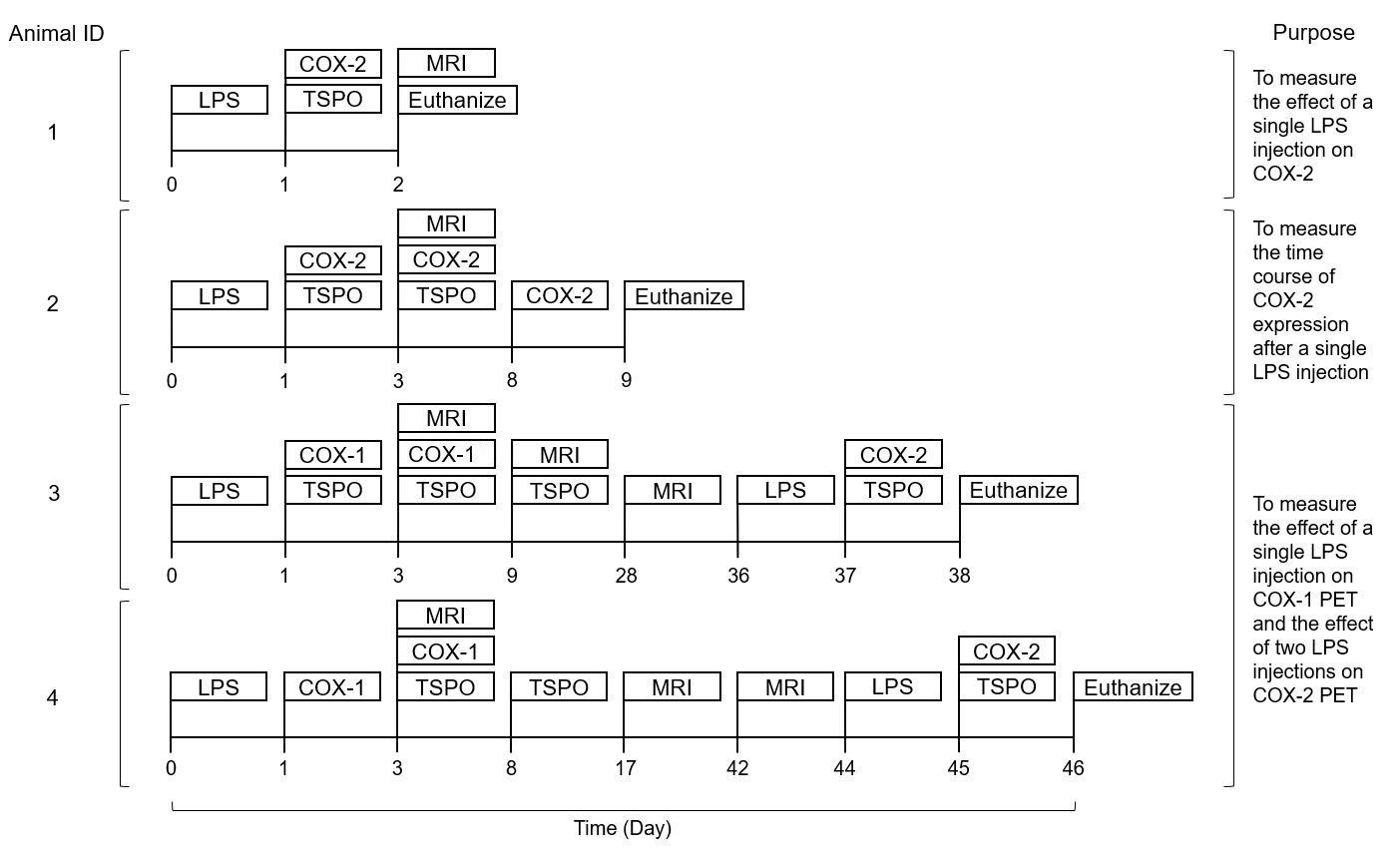
Supplementary Figure S1.** Timeline of the scans performed after lipopolysaccharide (LPS) injection. Monkeys 1 and 2 received one LPS injection, and Monkeys 3 and 4 received two LPS injections with the second injection being more than a month after the first injection. At the end of the study, animals were euthanized for in vitro measurements. The positron emission tomography (PET) scans for cyclooxygenase-1 (COX-1) and COX-2 included two in one day, before and after injection of a blocking agent.


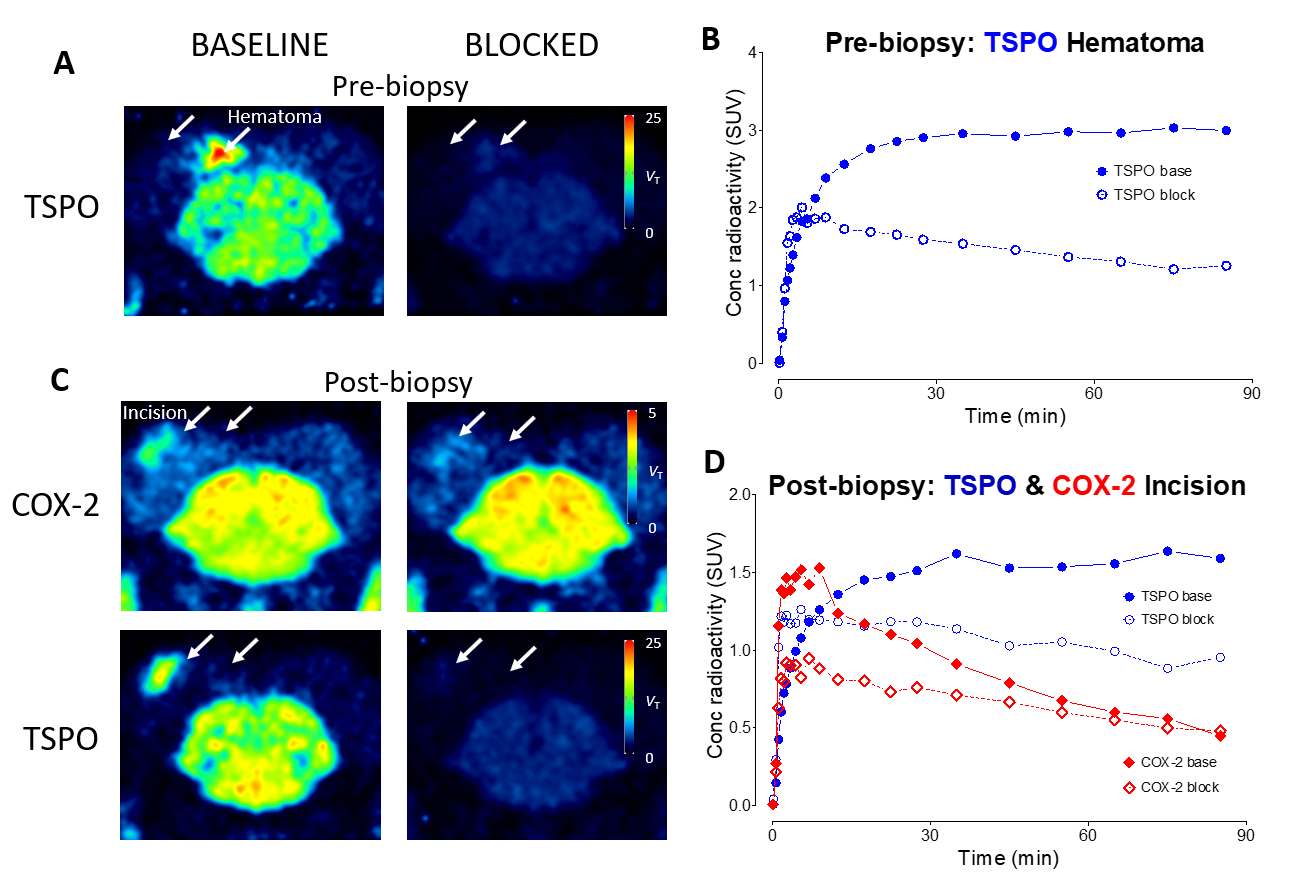


**Supplementary Figure S2**. Incidental finding in skull muscle of a mass with a positive and blockable translocator protein (TSPO) signal (A & B). Biopsy of the mass showed it to be a resolving hematoma. Imaging with [^11^C]MC1 three weeks after the biopsy showed that the cyclooxygenase-2 (COX-2) signal in the lesion had resolved, but the incision for the biopsy was positive for COX-2. Four additional weeks later (that is, almost nine weeks after the initial incidental PET scan), we scanned for TSPO again and found, like for COX-2, that the lesion was negative but that a clear line of TSPO positivity, blockable by PK11195, overlay the incision. In addition, uptake of both radioligands could be blocked; that is, there was specific binding to the two targets (C & D). In all PET images, the locations of the hematoma and the incision are marked by two white arrows.

**
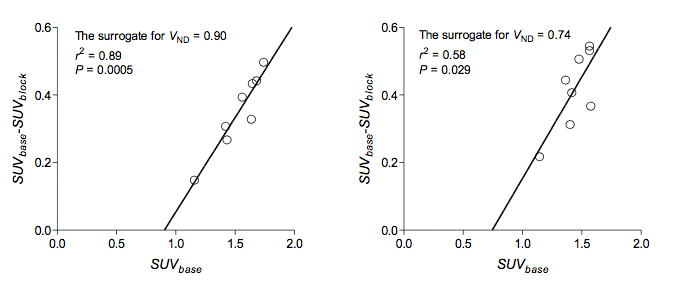
**

**Supplementary Figure S3.** Modified Lassen plots to determine cyclooxygenase-2 (COX-2) occupancy and the surrogate for non-displaceable uptake (*V*_ND_) of [^11^C]MC1 in the brains of the two patients with rheumatoid arthritis. Each point represents a brain region from a single patient scanned twice in one day: at baseline and after enzyme blockade by celecoxib (400 mg). The slope of the straight-line fit provides the occupancy. The x-intercept provides the surrogate for *V*_ND_.
